# Polyphyllin I Inhibits *Propionibacterium acnes*-Induced IL-8 Secretion in HaCaT Cells by Downregulating the CD36/NOX1/ROS/NLRP3/IL-1*β* Pathway

**DOI:** 10.1155/2021/1821220

**Published:** 2021-09-21

**Authors:** Shuyun Yang, Ying Jiang, Xiuqin Yu, Liping Zhu, Lu Wang, Jingzhu Mao, Miaomiao Wang, Naihui Zhou, Ziliang Yang, Ying Liu, Tingting Zhu

**Affiliations:** ^1^Department of Dermatology, Baoshan People's Hospital, Baoshan, Yunnan, China; ^2^Department of Dermatology, First Affiliated Hospital of Soochow University, Suzhou, Jiangsu, China; ^3^Department of Laser and Aesthetic Medicine, Department of Plastic and Reconstructive Surgery, Shanghai Ninth People's Hospital, Shanghai Jiao Tong University School of Medicine, Shanghai 200011, China

## Abstract

Acne vulgaris (AV) is a chronic skin disease involving inflammation of the pilosebaceous units. *Propionibacterium acnes* (*P. acnes*) hypercolonization is one pathogenic factor for AV. *P. acnes* that triggers interleukin-1*β* (IL-1*β*) by activating the pyrin domain-containing 3 protein (NLRP3) inflammasome of the NOD-like receptor family in human monocytes. Reactive oxygen species (ROS) acts as a trigger for the production of IL-8 and activates theNLRP3 inflammasome. IL-8 promotes the metastasis and multiplication of different cancerous cells, whereas keratinocyte proliferation and migration contribute to the progression of AV. A steroidal saponin called polyphyllin I (PPI) that is extracted from *Paris polyphylla*'s rhizomes has anti-inflammatory properties. This study investigates the regulatory role of *P. acnes* in the secretion of IL-8 mediated by the CD36/NADPH oxidase 1 (NOX1)/ROS/NLRP3/IL-1*β* pathway and the effects of PPI on the CD36/NOX1/ROS/NLRP3/IL-1*β*/IL-8 pathway and human keratinocyte proliferation and migration. HaCaT cells were cultured and stimulated with 10^8^ CFU/ml of *P. acnes* for 0, 6, 12, 18, 24, 30, and 36 hours. *P. acnes* induced IL-8 secretion from HaCaT cells via the CD36/NOX1/ROS/NLRP3/IL-1*β* pathway. PPI inhibited the CD36/NLRP3/NOX1/ROS/IL-8/IL-1*β* pathway and HaCaT cell proliferation and migration. PPI alleviates *P. acnes*-induced inflammatory responses and human keratinocyte proliferation and migration, implying a novel potential therapy for AV.

## 1. Introduction

The skin is the primary stress perceiving organ; hence, it is prone to inflammatory dermatoses [1, 2]. Acne vulgaris (AV) is a chronic skin disease that involves the inflammation of the pilosebaceous unit that affects adults as well as teens, but it is not fatal. However, affected individuals may be socially maladjusted due to severe acne and ongoing psychological effects [3]. Conventional treatments include hormonal therapy, oral antibiotics, and isotretinoin, but these strategies have different side effects including chapped lips and teratogenicity that limit isotretinoin medication [4]. In addition, isotretinoin is also known for causing inflammatory bowels in adolescents and children [5], and it is also reported to cause depression [6]. Drug resistance is another major concern due to the long-term and extensive use or overuse of antibacterial drugs [7]. Therefore, a more effective and safer AV therapy targeted to all age groups is in demand.

This study is aimed at exploring the underlying molecular and cellular mechanisms regarding the pathogenesis of AV. Previous studies have revealed that the hypercolonization *of Propionibacterium acnes* (*P. acnes*) is one mechanism of AV pathogenesis [8, 9]. *P. acnes* is a rod-shaped, anaerobic, Gram-positive pathogen mostly inhabitates the deep microaerophilic parts of healthy follicles, where it comes into contact with follicular keratinocytes and cells in the proximal part of the sebaceous duct. Clinical isolates of *P. acnes* (889) are capable of inducing primary human epidermal keratinocyte growth in vitro [10]. In addition, *P. acnes* mediates keratinocyte proliferation and elevates inflammation in the region where it resides, making it a key factor in causing inflammatory acne lesions and the development of microcomedos in the initial acne stages [11, 12].

The NOD-like receptor family, pyrin domain-containing 3 protein (NLRP3) inflammasome contains a variety of sensor molecules including NLRP3, the adaptor protein apoptosis-associated speck-like protein containing a CARD (ASC), and procaspase-1. NLRP3 has a pyrin domain (PYD) and an ASC that harbors both PYD and CARD domains. After its activation, NLRP3 interaction with ASC occurs through PYD and CARD domains of procaspase-1 to form the NLRP3-ASC-procaspase-1 complex, which is also referred to as the NLRP3 inflammasome [13]. By promoting caspase-1 activation, the NLRP3 inflammasome enhances the proteolytic maturation of the proinflammatory cytokine interleukin-1*β* (IL-1*β*) to facilitate its attachment to the IL-1 receptor [14]. IL-1*β* further induces the expression of the canonical IL-1 target gene interleukin-8 (IL-8) in human macrophages [15] and human endometrial stromal cells [16]. *P. acnes* activates IL-8 secretion by interacting with toll-like receptor 2 and toll-like receptor 4 (TLR-2 and TLR-4) present on the surface of human epidermal keratinocytes, contributing to the development of inflammatory lesions [10, 17]. Therefore, the assembly of NLRP3 inflammasome promotes caspase-1-mediated IL-1*β* secretion [18]. Previous studies show that *P. acnes* activates NLRP3 inflammasome and caspase-1 which promotes IL-1*β* secretion in human monocytes and sebocytes [19, 20]. This indicates the role of *P. acnes* in activating the NLRP3 inflammasome-mediated IL-8 secretion in human keratinocytes.

*P. acnes* activates NADPH oxidase 1 (NOX1) that triggers the release of reactive oxygen species (ROS) and secretion of IL-8 which is an essential proinflammatory cytokine in the pathogenesis of AV, by recognizing the thrombospondin receptor (CD36) on the surface of human keratinocytes (HaCaT cells) [21]. CD36 and other scavenger receptors bind to a range of microbial and endogenous cargoes and mediate their internalization [22, 23]. Several of these cargoes activate NLRP3 inflammasome. Based on this fundamental data, CD36 is likely to activate NLRP3 inflammasome through NOX1 following *P. acnes* stimulation in human keratinocytes.

ROS serves as a trigger that activates the NLRP3 inflammasome, facilitating the pathological processes. External stimuli such as a bacterial infection may trigger the activation of NLRP3 inflammasome [24, 25], causing the release of cytokines and O_2_^−^ effectors, which leads to chronic sterile inflammation and tissue injury [26, 27]. If the ROS is more than their scavengers during the local inflammatory response, it results in the increasing intracellular and extracellular oxidative stress and IL-8 secretion [28, 29], bringing about the progression of AV [30, 31]. Additionally, IL-1*β* directly acts on keratinocytes to suppress the expression of functional protein and nitric oxide availability to further contribute to local hyperkeratosis [32, 33].

*Paris polyphylla* is a traditional medicinal herb widely known in the Shennongjia National Nature Reserve of China, which has a wide range of beneficial components that act against immune disorders, infectious diseases, cardiovascular diseases, and cancer [34–38]. One of the medicinal elements of *P. polyphylla* is polyphyllin I (PPI), which is a steroidal saponin derived from the rhizomes of *P. polyphylla*, and it is known for its anti-inflammatory properties. For example, PPI reduces the severity of collagen-induced arthritis by suppression of the nuclear factor kappa B (NF-*κ*B) pathway in macrophages that causes the suppression of the intracellular inflammatory response [39]. Our previous study also shows that PPI reduces the secretion of inflammatory cytokines, including tumor necrosis factor-*α* (TNF-*α*), interleukin-6 (IL-6), and IL-8, and suppresses the expression of NF-*κ*B, TLR-2, and the mitogen-activated protein kinase (MAPK) signal transduction pathways in HaCaT cells infected by *P. acnes* [40]. Furthermore, PPI has been found to possess preclinical anticancer efficacy by inhibiting proliferation and invasion of various cancer types, including gastric cancer [41], prostate cancer [42], and hepatocellular carcinoma [43]. However, the effect of PPI on IL-8 secretion through modulating the NLRP3 inflammasome is unknown.

We, therefore, aimed to elucidate the ability of *P. acnes* to regulate IL-8 secretion via the NLRP3/CD36/NOX1/ROS/IL-1*β* pathway and the effects of PPI on the NLRP3/CD36/NOX1/ROS/IL-8/IL-1*β* pathway and human keratinocyte proliferation and migration. Our results will further explain the role of *P. acnes* in the pathogenesis of AV and supply basic in vitro experimental data for the application of PPI in AV therapy.

## 2. Materials and Methods

### 2.1. Culturing and Treatment of Keratinocytes

HaCaT cells (^#^CRL-1624; ATCC, USA; 5 × 10^5^ cells/ml) were cultured in Dulbecco's minimal essential medium (DMEM; ^#^D0819, Sigma Aldrich, USA). Fetal bovine serum (10%) and penicillin-streptomycin (100 U/ml) were added to the medium as supplements followed by 24 h of incubation in an environment optimized at 37°C and 5% carbon dioxide (CO_2_). The culture was then substituted with a medium containing no serum and the indicated concentration of PPI (^#^HR138751; Hairui Chemical, China; 0.3, 0.6, and 0.9 *μ*g/ml). Dimethyl sulfoxide (DMSO; W387509, Merck, USA) was utilized to dissolve PPI, and the terminal concentration of DMSO was 0.1% in the study. Therefore, 0.1% DMSO was used as the solvent control in the experiments. After 30 min, the cells were cocultured with heat-inactivated *P. acnes* (ATCC No. 6919; ^#^23-003-857; Thermo Fisher, USA; 1.0 × 10^8^ CFU/ml) for 8 h.

### 2.2. Western Blot

Lysis of HaCaT cells was carried out using radioimmunoprecipitation assay (RIPA) and lysis buffer supplemented with phenylmethanesulfonyl fluoride (PMSF) after their isolation. The mixture was incubated for 30 minutes followed by centrifugation. The supernatant was collected after centrifugation, and a BioRad protein assay reagent was used to read the protein concentration. Lysates were denatured by thorough mixing with 5x loading buffer. An SDS-PAGE (10–12%) was run for the separation of the samples that were then blotted onto Millipore Immobilon®-P Transfer Membranes supplied by Billerica, MA, USA. A solution of Tris-buffer saline with 0.1% Tween 20 in 5% nonfat milk was used as a blocking agent, and then, the samples were incubated with primary antibodies and horseradish peroxidase-conjugated goat-anti-mouse (^#^ab205719; Abcam, USA) and goat-anti-rabbit (^#^ab6721; Abcam) secondary antibodies. The primary antibodies included anti-CD36 (^#^18836-1-AP; Proteintech), anti-NOX1 (^#^ab55831; Abcam), anti-NOD-like receptor family, pyrin domain containing-1 protein (NLRP1) (^#^ab98181; Abcam), anti-NLRP3 (^#^ab214185; Abcam), anti-NOD-like receptor family CARD domain containing 4 (NLRC4) (^#^06–1125; Merck Millipore), antiabsent in melanoma 2 (AIM2) (^#^ab186043; Abcam), anti-ASC (^#^AB3607; Merck Millipore), antiactive caspase-1 (^#^24232; Cell Signaling Technology), antiprocaspase-1 (^#^sc-56036; Santa Cruz Biotechnology, USA), anticleaved IL-1*β* (^#^83186S; Cell Signaling Technology), anti-pro-IL-1*β* (^#^ab9722; Abcam), and anti-GAPDH antibodies (^#^ab9485; Abcam). All the antibodies were diluted at a ratio of 1 : 1000, except the anti-GAPDH antibody, which was used at a dilution of 1 : 5000. The results were analyzed as fluorescent signals generated with the help of SuperSignal® West Pico Chemiluminescent Substrate (^#^34577; Thermo Fisher).

### 2.3. Enzyme-Linked Immunosorbent Assay (ELISA)

A 96-well plate was used to seed 5 × 10^3^ HaCaT cells which were then cultured at 37°C (5% CO_2_) according to the set protocols. The supernatant from cultures cells was collected, and the concentration of IL-8 was calculated by the human IL-8 ELISA kit (^#^KHC0081; Thermo Fisher) following the manufacturer's protocols. The absorbance reading was done at a wavelength of 450 nm using a microplate photometer supplied by Thermo Scientific (^#^VLBL00D1).

### 2.4. siRNA Transfection and Reagent Usage

Human CD36 siRNA, NOX1 siRNA, and NC siRNA were synthesized by GenePharma (China) (Table 1). HaCaT cells were plated in a dish of 6 cm in size (5 × 10^5^ cells) a day before transfection with the Lipofectamine 2000 transfection reagent (^#^12566014; Thermo Fisher). Separate dilution of fifteen microliters (10 nM) of siRNA and 20 *μ*l Lipofectamine 2000 in 500 *μ*l of DMEM were mixed after 5 minutes followed by an incubation period of 20 minutes at 25°C. Then, the mixture was poured into the cell culture dishes with 5 ml of DMEM. After 6 h, the medium was substituted by fresh medium, and cells were harvested after24 hours of transfection.

HaCaT cells were treated with diphenylene iodonium (DPI, NOX1 inhibitor; ^#^S8639; Selleck Chemicals, USA; 5 *μ*M), N-acetyl-l-cysteine (NAC, ROS inhibitor; ^#^S1623; Selleck Chemicals; 600 mM), anti-IL-1*β* antibody (^#^ACZ885, canakinumab, Novartis; 100 nM), or IL-1 receptor antagonist protein (IL-RA; ^#^280-RA; R&D Systems Inc., USA; 100 nM) for 24 h before the following experiments.

### 2.5. Detection of ROS Levels

Flow cytometry detected the intracellular ROS accumulation using the cell-permeable fluorogenic probe 2′, 7′-dichlorodihydrofluorescein diacetate (DCFH-DA; ^#^S0033; Beyotime, China). The supernatant was removed from cultured cells that were treated with different reagents, following incubation in the dark in a fresh medium and 10 *μ*M DCFH-DA at 37°C for 1 h. Afterwards, the media from the culture was removed, and cold PBS was used to wash the cells. Next, after harvesting the cells, the pallets were suspended in 500 *μ*l of PBS. Sample analysis was performed at 480 nm excitation wavelength and an emission wavelength of 525 nm by a flow cytometer supplied by FACScan.

### 2.6. EdU Proliferation Assay

To determine whether PPI affects HaCaT cell proliferation, an EdU incorporation assay was carried out. 48 hours after cell seeding, 10 *μ*M EdU was poured into the culture following incubation for some time followed by fixation using 4% paraformaldehyde in PBS for 15 minutes. EdU labeling with an azide derivative of Apollo 643 was performed using a Cell-Light™ EdU Apollo 643 In Vitro Imaging Kit (^#^C10310-2, RiboBio Co., Ltd., China). A 652 nm laser was used for the excitation of Apollo 643. Microscopic images were obtained with a FluoView FV1000 confocal laser scanning microscope (Olympus, Japan). ImageJ (National Institutes of Health, Bethesda, MD, USA) was used to get composite images.

### 2.7. Transwell Migration Assay

For the migration assay, HaCaT cells were exposed to *P. acnes* and PPI (0.9 *μ*g/ml) or the same volume of 0.1% DMSO, and then, 5 × 10^4^ cells were transferred into each transwell insert pore of about 8 *μ*m (^#^3422; Corning, USA). The cells were then incubated for 24 hours followed by the removal of cells adhered to the upper surface of the insert, while the ones that adhered to the lower surface were stained with crystal violet (0.2%). Afterwards, isopropanol was used to dissolve the migratory cells, and the wavelength at 595 nm was considered to detect their optical density.

### 2.8. Statistical Analysis

Data interpretation was performed by one-way analysis of variance, followed by the Bonferroni post hoc test for comparing the mean values of multiple groups using GraphPad Prism 6 (GraphPad Software, USA), and *p* < 0.05 was considered a statistically significant difference. The results were presented as the mean ± SEM.

## 3. Results

### 3.1. *P. acnes* Induces NLRP3 Inflammasome Activation and IL-8 Release in HaCaT Cells

**Figure 1:**
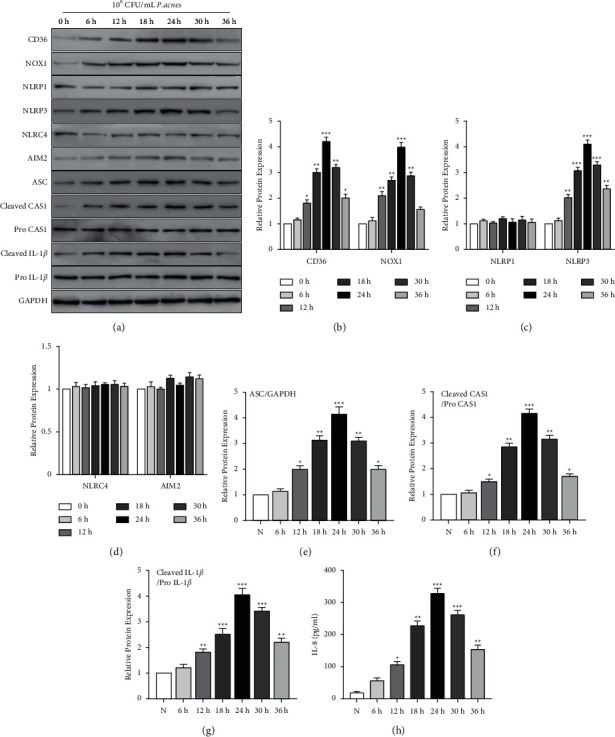
*P. acnes* inducing IL-8 release and the activation of NLRP3 inflammasome in HaCaT cells. (a) The CD36, NOX1, NLRP1, NLRP3, NLRC4, AIM2, ASC, active caspase-1, and cleaved IL-1*β* protein levels in HaCaT cells following 10^8^ CFU/ml *P. acnes* treatment detection done by Western blot. GAPDH was used as the loading control. CD36, NOX1 (b), NLRP1, NLRP3 (c), NLRC4, AIM2 (d), and ASC (e) relative protein levels compared to the GAPDH level, active caspase-1/pro-caspase-1, and cleaved IL-1*β*/pro-IL-1*β* are calculated by ImageJ. (h) ELISA was performed for the analysis of IL-8 protein levels in the culture supernatant of HaCaT cells. *n* = 5/each group. ^*∗*^*P* < 0.05 and ^*∗∗*^*p* < 0.01 vs. 0 h group.

The levels of CD36, NOX1, NLRP3, ASC, active caspase-1, and cleaved IL-1*β* protein were elevated in HaCaT cells after stimulation by *P. acnes*, while NLRP1, NLRC4, and AIM2 protein level showed no change (Figures 1(a)–1(g)). In addition, *P. acnes* also enhanced the secretion of IL-8 from HaCaT cells (Figure 1(h)). The results indicate that *P. acnes* induces the activation of NLRP3 inflammasome in HaCaT cells.

### 3.2. *P. acnes* Activates the CD36/NOX1/ROS/NLRP3/IL-1*β*/IL-8 Pathway in HaCaT Cell Line

**Figure 2:**
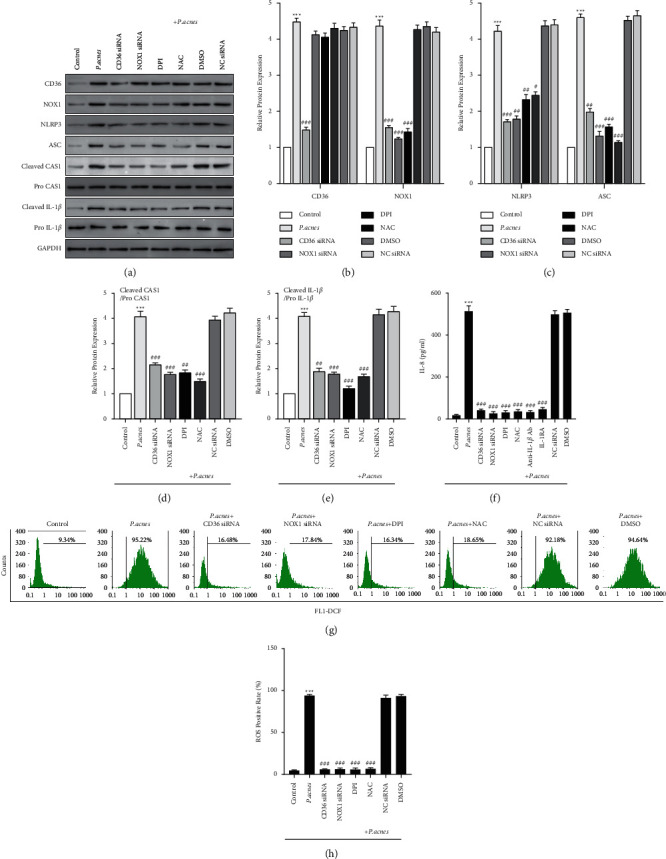
*P. acnes* inducing the CD36/NOX1/ROS/NLRP3/IL-1*β*/IL-8 pathway in HaCaT cells. (a) The CD36, NOX1, NLRP3, ASC, active caspase-1, and cleaved IL-1*β* protein levels in HaCaT cells following 10^8^ CFU/ml *P. acnes* treatment with CD36 siRNA, NOX1 siRNA, DPI, NAC, NC siRNA, or 01% DMSO exposure detected by Western blot. GAPDH is used as the loading control. CD36, NOX1 (b), NLRP3, and ASC (c) relative protein levels compared to GAPDH expression, active caspase-1/procaspase-1 (d), and cleaved IL-1*β*/pro-IL-1*β* (e) calculated by ImageJ. (f) The IL-8 protein levels in the culture supernatant of HaCaT cells determined by ELISA. (g) Cytofluorometric profiles representing the distribution of HaCaT cells after staining with DCFH-DA. (h) ROS positive ratio calculated by cytometry flow. *n* = 5/each group. ^*∗∗*^*P* < 0.01 vs. control group. ^#^*P* < 0.05 vs. *P. acnes* treatment group.

To choose the optimal siRNA for CD36 or NOX1 knockdown, transfection of HaCaT cells was carried out by four types of CD36 siRNAs, NOX1 siRNAs, or NC siRNA each, showing that CD36 siRNA2 and NOX1 siRNA2 were the most efficient (Supplementary Figures 1 and 2). CD36 siRNA decreased the NOX1 protein level, while NOX1 siRNA did not affect the protein levels of CD36. NLRP3, ASC, and active caspase-1, and cleaved IL-1*β* protein levels were downregulated by CD36 siRNA, NOX1 siRNA, DPI, and NAC (Figures 2(a)–2(e)). Similarly, IL-8 secretion was also inhibited by CD36 siRNA, NOX1 siRNA, DPI, NAC, anti-IL-1*β* Ab, and IL-RA (Figure 2(f)); this shows that the IL-8 secretion activates the NLRP3 inflammasome and triggers the production of IL-1*β*. Interestingly, CD36 siRNA, NOX1 siRNA, DPI, and NAC hindered ROS production (Figures 2(g) and 2(h)), indicating that oxidative stress promotes the activation of NLRP3 inflammasome in HaCaT cell line. The results suggested that *P. acnes* activated the pathways of NLRP3/CD36/IL-1*β*/NOX1/ROS/IL-8 in HaCaT cells.

### 3.3. PPI Inhibits the *P. acnes-*Induced CD36/NOX1/ROS/NLRP3/IL-8/IL-1*β* Pathway in HaCaT Cells

**Figure 3:**
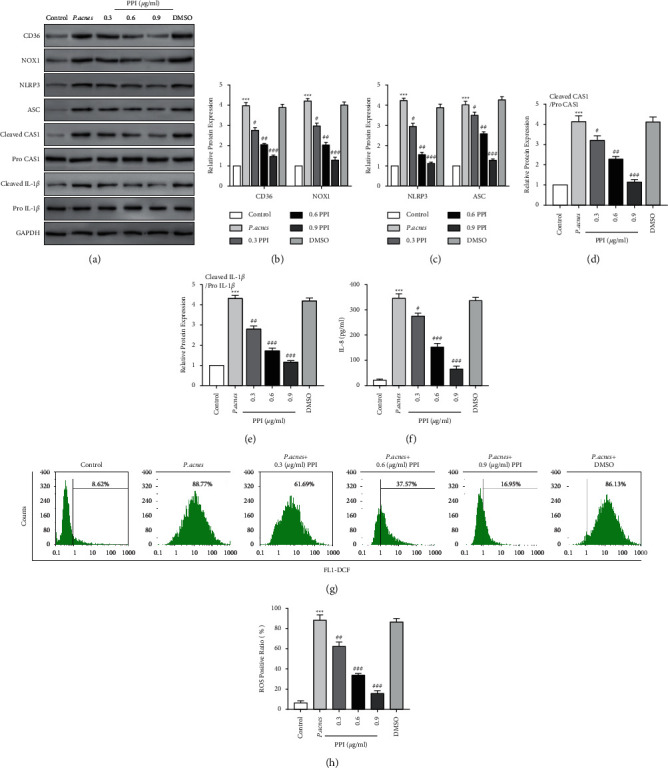
PPI inhibiting the *P. acnes*-induced CD36/NOX1/ROS/NLRP3/IL-8 pathway in HaCaT cells. The CD36, NOX1, NLRP3, ASC, active caspase-1, and cleaved IL-1*β* protein levels in HaCaT cells following 10^8^ CFU/ml *P. acnes* treatment with PPI at different concentrations (0.3, 0.6, and 0.9 *μ*g/ml) or 0.1% DMSO exposure detected by Western blot. GAPDH is used as the loading control. CD36, NOX1 (b), NLRP3, and ASC (c) relative protein levels compared to GAPDH expression, active caspase-1/procaspase-1 (d), and cleaved IL-1*β*/pro-IL-1*β* (e) calculated by ImageJ. (f) The IL-8 protein levels in the culture supernatant of HaCaT cells determined by ELISA. (g) Cytofluorometric profiles representing the distribution of HaCaT cells after staining with DCFH-DA. (h) ROS positive ratio calculated by cytometry flow. *n* = 5/each group. ^*∗∗*^*P* < 0.01 vs. control group. ^#^*P* < 0.05 vs. *P. acnes* treatment group.

Previous research reports the inhibitive properties of PPI in the *P. acnes*-induced HaCaT cell inflammatory response, such as elevated TLR2 levels and expression of inflammatory cytokines such as TNF-*α*, IL-6, and IL-8 [40]. Therefore, in this study, we investigated the ability of PPI to influence the *P. acnes*-induced CD36/NOX1/ROS/NLRP3/IL-1*β*/IL-8 pathway. CD36, NOX1, NLRP3, ASC, active caspase-1, and cleaved IL-1*β* protein levels were downregulated by different concentrations of PPI (Figures 3(a)–3(e)). PPI also downregulated ROS production in HaCaT cells (Figures 3(g) and 3(h)) and inhibited IL-8 secretion (Figure 3(f)). The above results suggested that PPI inhibited the *P. acnes*-induced CD36/NOX1/ROS/NLRP3/IL-1*β*/IL-8 pathway in HaCaT cells.

### 3.4. PPI Inhibits HaCaT Cell Proliferation and Migration

**Figure 4:**
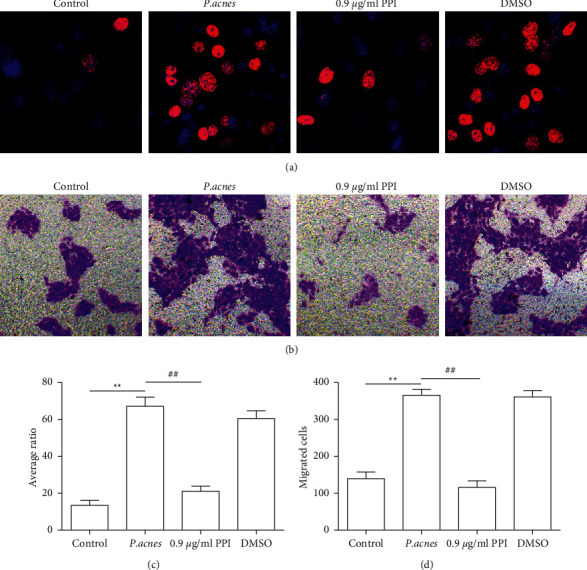
PPI inhibiting proliferation and migration of HaCaT cells. (a) The proliferation of HaCaT cells following *P. acnes* treatment with PPI (0.9 *μ*g/ml) or 01% DMSO exposure detected by the EdU incorporation assay. The red color represents EdU positive, and the blue color represents DAPI (nucleus). The average ratio of EdU positive HaCaT cells is calculated by ImageJ. (b) The migration of HaCaT cells following *P. acnes* treatment with PPI (0.9 *μ*g/ml) or 0.1% DMSO exposure detected by the transwell assay. (d) The average number of migrated HaCaT cells calculated by ImageJ. *n* = 5/each group. ^*∗∗*^*P* < 0.01 vs. control group. ^##^*P* < 0.01 vs. *P. acnes* treatment group.

**Figure 5:**
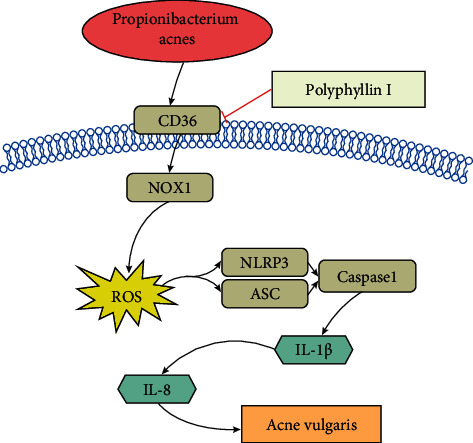
The schematic diagram showing PPI inhibited the *P. acnes-*induced CD36/NOX1/NLRP3/ROS/IL-1*β*/IL-8 pathway and HaCaT cell proliferation and migration.

Keratinocyte proliferation and migration contribute to the progression of AV [44, 45]. Moreover, PPI exhibits inhibitory effects on human ovarian cancer HO-8910PM cell growth, promotes apoptosis, and inhibits cell migration [46]. Therefore, the PPI effect of HaCaT cell proliferation and migration was evaluated. Consistent with its previously reported roles, PPI inhibited HaCaT cell proliferation (Figures 4(a) and 4(c)) and migration (Figures 4(b) and 4(d)) compared to those of the *P. acne*'s group. The data suggested that PPI might alleviate AV by downregulating keratinocyte proliferation and migration. The mechanism diagram showed that PPI inhibited *P. acnes*-induced CD36/NOX1/ROS/NLRP3/IL-1*β*/IL-8 pathway and HaCaT cell proliferation and migration (Figure 5).

## 4. Discussion

Human keratinocytes are cultured frequently for in vitro studies of and understanding of their inflammatory responses and immunological role. However, due to variable results from different donors, short culture time and passage variations may cause difficulty in interpreting the collected data. HaCaT cells are a sustainable cell line of spontaneously immortalized human keratinocytes. The previous study highlights the influence of cell density, different concentrations of Ca^2+^ ions in the medium, and the presence of serum at different levels on the secretion of proinflammatory mediators by these cells. Moreover, HaCaT cells survived in the low Ca^2+^ ionic medium and showed 80% resemblance to normal keratinocytes in their cytokine secretion suggesting that HaCaT cells are useful anti-inflammatory therapeutic agents for the investigation of dermatological ailments [47]. Additionally, HaCaT has been widely used for the research of acne vulgaris, such as the molecular mechanism study [48, 49], and potential drug screen [50, 51]. Therefore, HaCaT cells were used for this research.

Herein, we found that *P. acnes* activated the NLRP3 inflammasome in human keratinocytes. The NLRP3 inflammasome is a molecular platform that assembles in response to various stimuli, including excessive ROS levels, and has been extensively studied [52, 53]. *P. acnes* can produce ROS during the infection process [54, 55]. Therefore, whether *P. acnes* activates the NLRP3 inflammasome in human keratinocytes by upregulating intracellular ROS needs further investigation.

The combined effect of tumor necrosis factor-*α* and interleukin-17 enhances ROS release and improves NOX1 activity which in turn triggers the expression of lipocalin-2 (LCN-2) by controlling the expression of a major LCN-2 inducer called IkappaBzeta (I*κ*B*ζ*). In addition, mice models that were deficient in NOX1 had lowered levels of LCN-2 production and colon damage during TNBS-induced colitis [3]. Furthermore, UVA activates NOX1-based NADPH oxidase to release ROS that further stimulates the synthesis of prostaglandin E2. Hence, NOX1 presents as an appropriate target for the components that are designed to protect from UVA-induced skin injury [56]. Moreover, the NOX1 gene is knocked down using RNA interference for the confirmation of NOX being the major source of O_2_^•−^ in keratinocytes infected by *P. acnes*. The results showed that NOX1A-siRNA reduced significantly the production of O_2_^•−^*P. acnes*-transfected keratinocytes, with almost 100% inhibition rate after stimulation for 3 hours [21]. Therefore, previous research as well as our currents experiments support that NOX1 is the main source of *P. acnes*-induced oxidative stress in HaCaT cells.

ROS triggers not only the NLRP3 inflammasome but also the downstream effector molecule of this inflammasome. It is clearly understood that the ROS initiated oxidative stress and is directly or indirectly responsible for causing AV [31, 57]. Thus, antioxidant therapies have been applied for AV and have shown the expected efficacy [58, 59]. For example, topical or oral zinc, the vitamin C precursor sodium ascorbyl phosphate, and nicotinamide were reported to be effective against acne in multiple studies [60, 61]. Whether PPI plays an antioxidant role in human keratinocytes must be elucidated in future research.

In the previous study, researchers have identified that caspase-1, ASC, and NLRP3 knockdown or knockout inhibits *P. acnes-*induced IL-1*β* production in acne [33]. IL-1*β* drives inflammatory responses in *Propionibacterium acnes* both in vitro and in vivo. *P. acnes* activates the NLRP3 inflammasome of monocyte-macrophages and promotes the processing and secretion of IL-1*β*. Additionally, ultraviolet irradiation stimulates the NLRP3 inflammasome activation in keratinocytes [62]. Therefore, we did not perform similar experiments to confirm that *P. acnes* induces the NLRP3/ASC/caspase-1/IL-1*β* pathway in keratinocytes. Upon the stimulation of pattern recognition receptors, keratinocytes secrete IL-1*β* that causes rapid initiation of the immune response, leading to the expression of other cytokines, including IL-8 [63]. In the present study, we also found that IL-1*β* promoted IL-8 secretion by HaCaT cells following *P. acnes* treatment.

In summary, *P. acnes* induced the production of ROS, activation of the NLRP3 inflammasome, and IL-8 release in HaCaT keratinocytes, while PPI inhibited ROS production, NLRP3 activation, IL-8 secretion, and HaCaT keratinocyte proliferation and migration, therefore suggesting a potential treatment strategy for AV. Certainly, there are some limitations of our study that were worthy of being addressed in future studies; for example, the detailed mechanisms of NLRP3 inflammasome activation and human AV lesion observation should be studied in the future.

## Figures and Tables

**Table 1:** The sequence of each siRNA used in the study.

Name of siRNA	Sequence
CD36 siRNA1	5′-GCAAACAUGUUCAGAAGUC-3′
CD36 siRNA2	5′-CAUAGGACAUACUUGGAUA-3′
CD36 siRNA3	5′-GCAAGUUGUCCUCGAAGAA-3′
CD36 siRNA4	5′-GGAAAGUCACUGCGACAUG-3′
NOX1 siRNA1	5′-GGUUAGGGCUGAAUGUUUU-3′
NOX1 siRNA2	5′-CUGCCUACAUACAGCUAUU-3′
NOX1 siRNA3	5′-GACAAAUACUACUACACAA-3′
NOX1 siRNA4	5′-UGAGAAAGCAAUUGGAUCA-3′
NC siRNA	5′- UAACAAUGAGAGCACGGCTT-3′

## Data Availability

The data used to support the findings of this study are available from the corresponding author upon request.

## Supplementary Materials

Supplementary Materials Supplementary Figure 1: HaCaT cells transfected by CD36 siRNA, NOX1 siRNA, or NC siRNA for 24 h. Then, the cell total protein lysates were extracted. The CD36 siRNA transfection efficiency was determined by Western blot (A) and statistical analysis (B). The NOX1 siRNA transfection efficiency was determined by Western blot (C) and statistical analysis (D). Supplementary Figure 2: HaCaT cells with PA treatment transfected by CD36 siRNA, NOX1 siRNA, or NC siRNA for 24 h. Then, the cell total protein lysates were extracted. The CD36 siRNA transfection efficiency was determined by Western blot (A) and statistical analysis (B). The NOX1 siRNA transfection efficiency was determined by Western blot (C) and statistical analysis (D).
