# Supplementary figures and images for: Polyphyllin I Inhibits Propionibacterium acnes-Induced IL-8 Secretion in HaCaT Cells by Downregulating the CD36/NOX1/ROS/NLRP3/IL-1β Pathway
Source: Evid Based Complement Alternat Med. 2021 Sep 21;2021:1821220. doi: 10.1155/2021/1821220 (PMC8481039; doi:10.1155/2021/1821220)

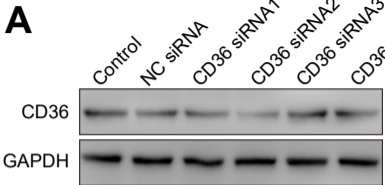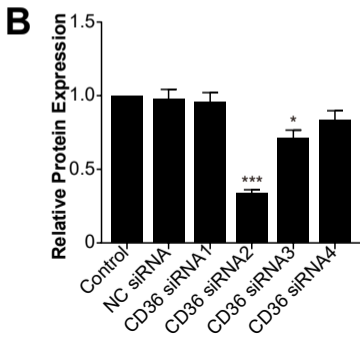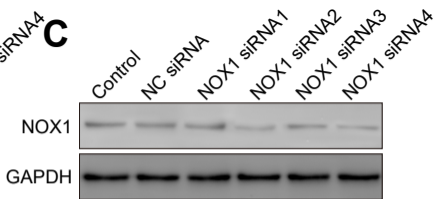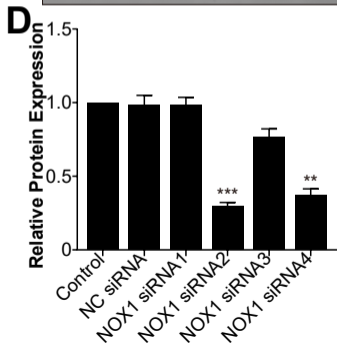

Supplement: Supplementary Materials — Supplementary Figure 1: HaCaT cells transfected by CD36 siRNA, NOX1 siRNA, or NC siRNA for 24 h. Then, the cell total protein lysates were extracted. The CD36 siRNA transfection efficiency was determined by Western blot (A) and statistical analysis (B). The NOX1 siRNA transfection efficiency was determined by Western blot (C) and statistical analysis (D). Supplementary Figure 2: HaCaT cells with PA treatment transfected by CD36 siRNA, NOX1 siRNA, or NC siRNA for 24 h. Then, the cell total protein lysates were extracted. The CD36 siRNA transfection efficiency was determined by Western blot (A) and statistical analysis (B). The NOX1 siRNA transfection efficiency was determined by Western blot (C) and statistical analysis (D). [file 1821220.f1.zip › 1821220.f1/supplementary figure1.pdf]

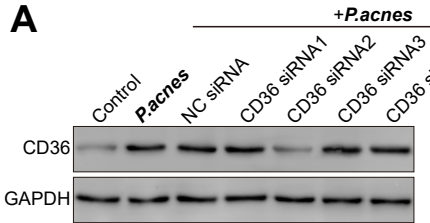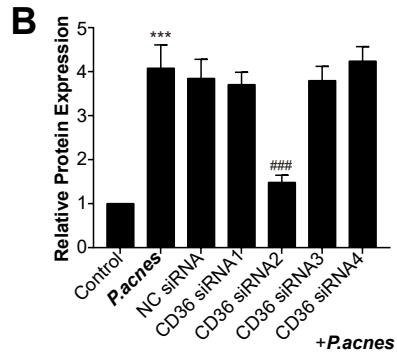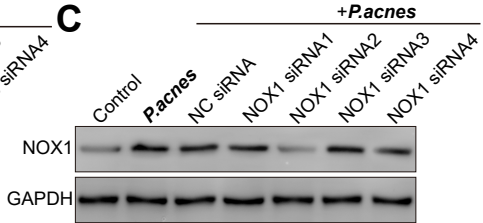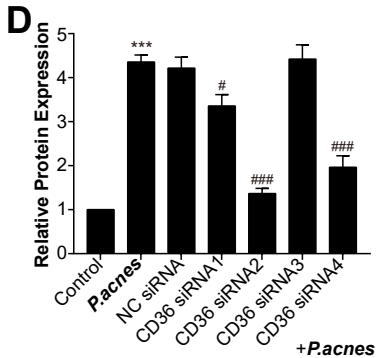

Supplement: Supplementary Materials — Supplementary Figure 1: HaCaT cells transfected by CD36 siRNA, NOX1 siRNA, or NC siRNA for 24 h. Then, the cell total protein lysates were extracted. The CD36 siRNA transfection efficiency was determined by Western blot (A) and statistical analysis (B). The NOX1 siRNA transfection efficiency was determined by Western blot (C) and statistical analysis (D). Supplementary Figure 2: HaCaT cells with PA treatment transfected by CD36 siRNA, NOX1 siRNA, or NC siRNA for 24 h. Then, the cell total protein lysates were extracted. The CD36 siRNA transfection efficiency was determined by Western blot (A) and statistical analysis (B). The NOX1 siRNA transfection efficiency was determined by Western blot (C) and statistical analysis (D). [file 1821220.f1.zip › 1821220.f1/supplementary figure2.pdf]
